# Renal Cell Carcinoma: A Complex Therapeutic Challenge in the Elderly

**DOI:** 10.7759/cureus.26346

**Published:** 2022-06-26

**Authors:** Mashood Iqbal

**Affiliations:** 1 Internal Medicine, Jinnah Medical College Hospital, Karachi, PAK

**Keywords:** renal cell carcinoma (rcc), older-aged patients, elderly patients, genetic mutations, surgical removal, mortality rate, malignant tumor

## Abstract

An increase in age and the occurrence of renal cell carcinoma have been positively correlated. A strict therapeutic protocol for early diagnosis, screening, prevention, and population awareness needs to be well-established as a rationale to approach the morbidity at a treatment-eligible phase in the aged. Genetic predisposition appears to have a minor role in the disease pathology. Imaging modalities, providing high-resolution images of the tumor, have undoubtedly benefitted the diseased subset in aiding the diagnosis, however, a preliminary guideline protocol for its early implication in concordance with the initial symptoms needs to be adopted. Burdening of the geriatric age group by concomitant co-morbidities further deteriorates the devastating effects of the primary tumor, which, in total, appear to evolve as a final, complex stage of the illness in the majority, leading to an eventual high mortality rate. Despite being a challenging task for managing the tumor, age should not be considered the sole treatment barrier for approaching the disease.

## Introduction and background

The management of renal cell cancer in the elderly has been highlighted as a complex physician task where the occurrence of morbidity has been strongly correlated to an increase in age [1-2]. Malignant tumors of the kidney account for more than 2% of cancer incidence with more than 80% of kidney cancers arising in the renal parenchyma and the remainder in the renal pelvis [3]. Renal cell carcinoma (RCC) has the highest mortality rate of genitourinary cancers, with a steady rise in its incidence claiming it to specifically involve a higher proportion of the elderly population [4-6]. New studies have also emphasized the role of obesity, a co-morbidity, in the development of the neoplasm [7]. This cancer has been primarily addressed throughout the literature as a disease of the elderly, typically presenting in the sixth and seventh decades of life [8]. Advanced age and co-morbidities have been clearly regarded as treatment barriers where the co-morbidities as a domain are bound to be more prevalent in the aged in comparison to the young, with surgical removal of the tumor being the only potentially curative therapy in early-stage RCC. The fundamental need for cost-effective screening programs with geriatric counseling sessions due to an increased ratio of co-morbidity-related peri-operative mortality has been highlighted to make a difference in the future of renal cell cancer therapeutics [2,5-6].

## Review

RCC symptomatology, statistics, and demographic data

Renal cell carcinoma evolves as the ninth most common cancer worldwide, with an overall five-year survival rate residing at 75.2% along with the morbidity being clearly labeled as highly fatal [7,9]. The National Cancer Institute emphasizes an intriguing five-year survival pattern at 93% with the tumor being localized. Their statistics also reveal an estimated discovery of new cases amounting to 79,000 in 2022, securing its eighth position among the top 10 most common neoplasms encountered [10]. The global incidence rate of diagnosis occurs at 2% with an increase in its overall worldwide occurrence. Prior epidemiological data have clearly revealed an estimated relative risk of the disease at 1.7 for men in comparison to women; males diagnosed 1.7 times more likely than females. The latter gender disparity was based according to the 2018 GLOBOCAN Data where 403,000 were annually diagnosed with neoplastic lesions in the kidney [11]. Prior published data has also claimed RCC as the third most common urogenital cancer worldwide with an incidence of about 200,000 cases worldwide annually where the latter is expressed per 100,000 population [1,12]. Diagnosis of the ailment continues to preside in the literature as being incidental in occurrence in the majority of the cases encountered. The stage at which the incidental discovery is determined strongly correlates with the eventual prognosis and overall survival justified by the statistics of a disseminated renal carcinoma having a five-year survival rate of merely 12% [11].

Age, in specific, has been graded as a non-modifiable entity where 75 years and older subset have been depicted as having the largest rise in the incidence of the disease [11]. In contrast to this robust correlation of the disease with increasing age, the Canadian Journal of Urology has also recently concluded that renal cell carcinoma if diagnosed in the young display a prolonged overall survival rate and decreased recurrence than in adults, with a total of 44,673 cases of renal cell carcinoma identified from 1975 to 2016, out of which a startling 41,812 cases were from the conventional old age group [13]. This evidently clarifies the high-risk population justifying the ultimate neoplastic burden on the aged subset. The overall incidence of RCC in the young and pediatric age group, however, appears to be scarce in the literature [14].

The frequently encountered symptomatic triad of flank pain, hematuria, and abdominal mass presents in merely 10-15% of patients and precisely indicates an advanced stage of the disease thereby signifying the disadvantage of diagnosing the tumor primarily on the basis of its symptoms [11,15]. Symptomatology, therefore, cannot be graded as an effective diagnostic tool in aiding the diagnosis. Despite carcinoma playing a pivotal role in the high mortality per the statistics, age has also been stated to not be considered a sole obstruction when considering the therapeutic strategy of the morbidity [16].

Subtypes, early diagnosis, and evaluation of RCC

The cardinal histological variants of RCC include clear cell, papillary, and chromophobe with 75%, 20%, and 5% being their percentages of occurrence, respectively [17]. Diagnosis by ultrasound, MRI, and CT imaging modalities have been proven to be beneficial in the early detection of RCCs [11,18]. Early detection has also been declared as the key concept to a favorable prognosis and outcome, however, a majority of the cases being diagnosed are incidental, and 30% of the cases metastatic upon confirmation of the ailment, which embarks upon the need for a productive intervention [17,19]. The Journal of Urologic Radiology suggests that RCC presents with a cystic morphology mainly comprising unilocular or multilocular cystic appearances. It highlights the effectiveness of CT imaging as a positive tool to detect calcifications that occur as part of the tumor oncogenesis [20]. Another survey on 36 patients with renal cell carcinoma also claimed papillary tumors to appear hyperechoic on ultrasound and the visualization of a tumor capsule on ultrasound clearly enables imaging techniques to preside as pioneers for ultimate diagnosis [21]. Considering the above features of the carcinoma, it is also pertinent to mention that the neoplasm is frequently diagnosed in elderly patients as proven by Bladinger L et al. [22].

Considering the non-elderly statistics, Mariana M et al. have evidently claimed the scarcity of the neoplasm in the young age group [14]. The 2020 cancer statistics for adolescents and young adults have highlighted a comparative analysis of the death rates from kidney and renal pelvis neoplasms from 2013 to 2017 in the 15-19 and 30-39-year age groups. The survey has explicitly stated the death rate to preside at less than 0.1 in the 15-19 and 0.3 in the 30-39 age groups, respectively. The latter data can be utilized to justify the hefty burden of the disease on the frail elderly subset [23]. This concept is further enhanced by 64 being the median age at diagnosis of RCC [24].

In accordance with what has been published, the concurrent rise in the aging population and the incidence of RCC predispose a clear challenging assignment for tumor approach and management in the aged [1]. A wide subset of elderly patients with a diagnosis of RCC presents with other major co-morbidities alongside cancer, causing them to have a poor functional and performance status alongside the established risk factors [6]. Such frail patients become poor candidates for clinical trials, as the results of their treatment become a challenge for interpretation. This ineligibility eventually leads to a lack of evidence-based guidelines for their treatment and poses oncologists with a difficult strategy to embrace in order to offer efficacious treatment to such patients [24]. Despite the pessimistic observations, an intriguing study, however, illuminates the concept of advanced age not being used as a criterion to deny curative management for renal cell carcinoma, hinting that the age tag should not be considered a solitary therapeutic barrier for RCC [5].

Flank pain, gross hematuria, and abdominal mass may represent the advanced stage of the disease course, however, metastatic cases have also been discovered to present with bone pain or persistent cough [15,25]. Natural non-modifiable factors need to be considered when dealing with the elderly population where, for instance, aging, is a complex process that affects every aspect of life. A literature review also establishes the pivotal role of pre-treatment performance status evaluation, which has been determined on the basis of several clinical trials, ultimately having a proven role in the prognosis. Despite keeping the above two factors into consideration, a thorough evaluation of co-morbidities and organ functional reserve appears obligatory. Co-morbidities, including hypertension, diabetes mellitus, cardiac diseases, cerebrovascular diseases, and respiratory diseases, have been well-documented in the RCC geriatric population [5].

Studies suggest that 30% of patients with RCC die because of conditions other than the latter. This highlights the effect co-morbidities can have alongside cancer, hence further burdening the frail subset. Considering that hemodialysis (HD), a widely used form of renal replacement therapy in the elderly, has been clearly linked with an increased risk of progression toward RCC. Similarly, the overall relative risk of RCC is 5-10-times higher in patients with end-stage renal failure [24]. In accordance with the use of HD, a comparative survey revealed the incidence of genitourinary cancer in patients who underwent HD versus the control group with a significant difference of 1.6% to 0.8%, respectively, emphasizing the pathological burden of the intervention to acquire the malignancy at a higher pace and conclude a steady relationship of hemodialysis toward the development of ultimate RCC [26].

Despite the rarity of renal cell tumors in adolescents, the preferred treatment of mainstay overall appears to involve surgical excision. However, a failure of the latter option or an underlying end-stage renal disease (ESRD) may result in the ultimate progression towards HD where the management of patients from all age groups appears as a challenging confrontation, with older HD patients being specifically vulnerable to die from other complications before even developing the malignancy [26-27]. An interesting study by Pan HC et al. concludes a higher risk of young with HD to develop cancer in comparison to the geriatric age group highlighting the mandatory need for an overall intensive and efficacious cancer screening protocol [24,26].

Primary tumor evaluation in developing countries, such as Pakistan, involves ultra­sound where the literature suggests that if the modality is executed on every pa­tient presenting with specific and nonspecific symp­toms in the urology outpatient department, RCCs can easily be diagnosed in the early stages for better survival outcomes. Hence, this provokes the need for regular physician follow-ups for a preliminary and efficacious confirmatory detection of RCC [24-25]. The disease has also been regarded as a financial burden on the health care setup, hence, this is an alarm to effectively acknowledge and deliver morbidity management effectively [28]. 

RCC treatment strategy

Surgical Intervention

When considering the treatment of the lethal morbidity, a study has strongly pointed out that the disease, if advanced, is highly resistant to systemic therapy [29]. Despite the resistance, the first interventional physician approach strategy that has been highlighted is the standard curative approach for localized RCC involving surgical excision with total nephrectomy. The positive role of this procedure for the elderly subset remains unclear; however, for the frail subset, a high risk of complications awaits their post-interventional phase [1,24,30]. The concept of an ideal balance between the risk and benefit of surgery needs to be addressed cautiously, again creating a complex therapeutic challenge for the physician [31].

Nephron-sparing surgical intervention has been identified as a cornerstone treatment, where in accordance with the National Comprehensive Cancer Network (NCCN) guidelines, all patients with RCC stages I-III fit for the surgical procedure must undergo nephrectomy (radical or nephron-sparing) as part of the treatment plan, implying surgical intervention as the primary curative procedure in the treatment strategy [7,24]. The geriatric or poor performance status patients still amenable to surgery primarily require nephron-sparing nephrectomy rather than radical nephrectomy, suggesting surgical excision as the dominant physician therapeutic approach for a better outcome in the older subset where merely age cannot be an exclusion criterion for treatment despite the higher risk involved in the former [24,32-33]. Authors also justify the implementation of laparoscopic surgery preferably in old-age patients as the rate of complications anticipated may be low. Studies also emphasize partial nephrectomy (PN) for solitary, small RCCs less than 4 cm in size alongside positively claiming the prognosis for surgical resection of small renal tumors as satisfactory with a five-year cancer-specific survival rate of 97% and 87% for T1a and T1b, respectively [1].

A retrospective analysis conducted from 1988 to 2005 revealed a startling number of 59,944 patients treated for RCC with surgery, out of which 4587 patients were aged greater than 80 years. Intriguingly, the survey clearly highlighted the burden of underlying debilitated and feeble functional status as a key factor in the progression of the disease towards mortality due to causes except for RCC [1]. This explicitly elevates the devastating effects of age on the RCC disease course, with a fundamental necessity to establish treatment prerequisites, guidelines, and a rationale predominantly related to the elderly subset. 

Ablative Therapy and Active Surveillance

Decreased life expectancy with associated co-morbidities provokes a detrimental effect and enlightens the utilization of management options such as ablative therapy or active surveillance [24]. Ablative modality comprises cryo or radiofrequency ablation, which could be proceeded percutaneously under local anesthesia, enabling a minimally invasive treatment option for elderly frail patients with co-morbidities who cannot be intervened by the conventional surgical approach [29,34]. The added advantage of the percutaneous ablative approach tends to involve an affording cost, less hospital stay duration, and a diminished rate of post-treatment complication phase in comparison to the conventional surgical modality [34]. Thermal ablation as a successful intervention has also been acclaimed as an analogous therapeutic option to the conventional surgical approach in the aged, prioritizing its key role in considering it as a treatment guideline precisely for the elderly [35]. Another prior scrutinization done on 84 tumors ablated demonstrated a five-year recurrence-free survival rate and a metastasis-free survival rate of 93% and 95%, respectively. These intriguing results enlighten a distinct physician approach that may be eventually considered as a rationale for the elderly subset [1].

Active surveillance evolves as an important observational tool, particularly in those patients with an observed slow growth rate of small renal masses. This monitoring can be highly successful where patients could be reassured that if progression occurs, a delayed operation can be proposed, with the same outcome as for immediate intervention. This can be mentioned as the least invasive yet effective therapeutic strategy particularly to be thoroughly considered in the elderly [1,22,36].

Metastasis and Immunotherapy

Metastatic RCC comprises 20-30% of patients with underlying RCC, which warrants a multidisciplinary approach. Surgical management includes cytoreductive nephrectomy in combination with metastasectomy of distant metastases. It is quite pertinent to mention the requirement of a satisfactory underlying functional status in order to acquire this treatment, especially for the older population. The positive role of this therapy, however, still remains unclear in the treatment protocol section of this literature review. The implications of Immunotherapy with interferon alfa and interleukin-2, which have been disregarded due to their potentially toxic and ineffective characteristics have been overcome by targeted therapies known to lead the treatment for metastasis [24,30].

From prolonged clinical trials, drugs, including sunitinib, Sorafenib, temsirolimus, bevacizumab, pazopanib, and everolimus have been found to be safe and effective in the elderly, frail, or in young and good performance status patients. The study, however, eventually concluded that newer targeted drugs should be offered to elderly patients with multiple co-morbidities, enhancing further treatment options in the aged [24]. Recent studies also quote an additional inclusion of improved quality of life and a cornerstone of treatment with the utilization of nivolumab with ipilimumab, specifically in poor-risk patients, which could possibly provide a major breakthrough in the quality management of the disease [37-38]. Nivolumab has also been regarded as having a pivotal role in treating pretreated cases of metastatic RCC exclusively in the aged [39].

Radiotherapy

To create a positive impact on the treatment of different tumor encounters, inoperable RCC can also be dealt with radiotherapy with surprisingly promising overall results. According to an interpretation of an analysis, 58 patients with inoperable or metastatic RCC received high-dose stereotactic radiotherapy (32Gy in four fractions, 40Gy in four fractions, or 45Gy in three fractions) where a stable disease was observed in 90% of patients with a local control rate of 90-98%. It has also been established that radiotherapy can be safely administered to elderly and frail patients and should be considered a therapeutic option whenever radical intervention is not applicable [24]. A conclusion could hence be generated where aged patients with no chance of curative resection can undoubtedly benefit from radiotherapy with positive long-term survival.

## Conclusions

Therapeutic management of renal cell carcinoma in the aged subset can now be achieved and is no longer an unsuccessful task. The classic triad of symptoms, including flank pain, hematuria, and abdominal mass, usually represent an advanced stage of RCC and are not reliable for a preliminary RCC diagnosis. The frail physique of the elderly subset is linked to higher mortality due to the underlying burden of co-morbidities. Regular and treatment-compliant patient follow-ups for an early tumor diagnosis and, eventually, better overall survival appears to be a primary key factor. Despite the cynical facts related to management and statistics, advanced age should not be contemplated as a sole barrier to surgery. Percutaneous ablation and immunotherapy with nivolumab have been established as an efficacious therapeutic strategy in the elderly subset for RCC. Nephron-sparing nephrectomy should be prioritized over radical nephrectomy, particularly in the aged population diagnosed with localized tumor configuration. Elderly and frail patients must be included in clinical trials in order to determine safe and effective treatment strategies and protocols for the future.

## References

[1] Quivy A, Daste A, Harbaoui A, Duc S, Bernhard JC, Gross-Goupil M, Ravaud A (2013). Optimal management of renal cell carcinoma in the elderly: a review. Clin Interv Aging.

[2] Fujita T, Hirayama T, Ishii D, Matsumoto K, Yoshida K, Iwamura M (2018). Efficacy and safety of sunitinib in elderly patients with advanced renal cell carcinoma. Mol Clin Oncol.

[3] Chow WH, Devesa SS, Warren JL, Fraumeni JF Jr (1999). Rising incidence of renal cell cancer in the United States. JAMA.

[4] Cairns P (2010). Renal cell carcinoma. Cancer Biomark.

[5] Berdjis N, Hakenberg OW, Novotny V, Froehner M, Wirth MP (2006). Treating renal cell cancer in the elderly. BJU Int.

[6] Capitanio U, Bensalah K, Bex A (2019). Epidemiology of renal cell carcinoma. Eur Urol.

[7] Turco F, Tucci M, Di Stefano RF (2021). Renal cell carcinoma (RCC): fatter is better? A review on the role of obesity in RCC. Endocr Relat Cancer.

[8] Zanardi E, Grassi P, Cavo A (2016). Treatment of elderly patients with metastatic renal cell carcinoma. Expert Rev Anticancer Ther.

[9] Jonasch E, Gao J, Rathmell WK (2014). Renal cell carcinoma. BMJ.

[10] (2022). The Surveillance Epidemiology, and End Results (SEER) Program. Cancer Stat Facts. Kidney and renal pelvis cancer. https://seer.cancer.gov/statfacts/html/kidrp.html.

[11] Padala SA, Barsouk A, Thandra KC (2020). Epidemiology of renal cell carcinoma. World J Oncol.

[12] Ather MH, Masood N, Siddiqui T (2010). Current management of advanced and metastatic renal cell carcinoma. Urol J.

[13] Liu F, Davaro F, Wong R, Siddiqui S, Hamilton Z (2022). Young age is associated with decreased recurrence for renal cell carcinoma. Can J Urol.

[14] Cajaiba MM, Dyer LM, Geller JI (2018). The classification of pediatric and young adult renal cell carcinomas registered on the children's oncology group (COG) protocol AREN03B2 after focused genetic testing. Cancer.

[15] Ata-ur-Rehman R, Ashraf S, Rahim J, Hussain N, Jamil MN, Tahir MM (2015). Clinical presentation of renal cell carcinoma. J Ayub Med Coll Abbottabad.

[16] Kanesvaran R, Le Saux O, Motzer R (2018). Elderly patients with metastatic renal cell carcinoma: position paper from the International Society of Geriatric Oncology. Lancet Oncol.

[17] Gray RE, Harris GT (2019). Renal cell carcinoma: diagnosis and management. Am Fam Physician.

[18] Escudier B, Porta C, Schmidinger M (2019). Renal cell carcinoma: ESMO Clinical Practice Guidelines for diagnosis, treatment and follow-up. Ann Oncol.

[19] Maher ER (2018). Hereditary renal cell carcinoma syndromes: diagnosis, surveillance and management. World J Urol.

[20] Dalla-Palma L, Pozzi-Mucelli F, di Donna A, Pozzi-Mucelli RS (1990). Cystic renal tumors: US and CT findings. Urol Radiol.

[21] Yamashita Y, Takahashi M, Watanabe O (1992). Small renal cell carcinoma: pathologic and radiologic correlation. Radiology.

[22] Baldinger L, Mehrazin R, Tomaszewski JJ, Uzzo RG (2014). Renal cell carcinoma: management in the elderly. Curr Geriatr Rep.

[23] Miller KD, Fidler-Benaoudia M, Keegan TH, Hipp HS, Jemal A, Siegel RL (2020). Cancer statistics for adolescents and young adults, 2020. CA Cancer J Clin.

[24] Rajer M (2012). Treatment of renal cell carcinoma in elderly and frail patients. Emerging research and treatments in renal cell carcinoma. InTechOpen.

[25] Naeem M, Khan MK, Ullah H, Khan MI, Izhar A, Alamgir K (2014). Pattern of presentation of renal cell carcinoma at Institute of Kidney Diseases, Peshawar. Journal of Postgraduate Medical Institute.

[26] Pan HC, Sun CY, Wu IW, Tsai TL, Sun CC, Lee CC (2019). Higher risk of malignant neoplasms in young adults with end-stage renal disease receiving haemodialysis: a nationwide population-based study. Nephrology (Carlton).

[27] Dreisin A, Matrana M (2016). Treating renal cell carcinoma in young adults: challenges and solutions. Clinical Oncology in Adolescents and Young Adults.

[28] Chien CR, Geynisman DM, Kim B, Xu Y, Shih YT (2019). Economic burden of renal cell carcinoma-part I: an updated review. Pharmacoeconomics.

[29] Penticuff JC, Kyprianou N (2015). Therapeutic challenges in renal cell carcinoma. Am J Clin Exp Urol.

[30] Wolchok JD, Motzer RJ (2000). Management of renal cell carcinoma. Oncology (Williston Park).

[31] Martini A, Cumarasamy S, Hemal AK, Badani KK (2019). Renal cell carcinoma: the oncological outcome is not the only endpoint. Transl Androl Urol.

[32] An JY, Ball MW, Gorin MA (2017). Partial vs radical nephrectomy for T1-T2 renal masses in the elderly: comparison of complications, renal function, and oncologic outcomes. Urology.

[33] Lv J, Song R, Cai H, Lu C (2019). Outcomes of laparoscopic radical nephrectomy for elderly patients with localized renal cell carcinoma. J BUON.

[34] Filippiadis D, Mauri G, Marra P, Charalampopoulos G, Gennaro N, De Cobelli F (2019). Percutaneous ablation techniques for renal cell carcinoma: current status and future trends. Int J Hyperthermia.

[35] Xing M, Kokabi N, Zhang D, Ludwig JM, Kim HS (2018). Comparative effectiveness of thermal ablation, surgical resection, and active surveillance for T1a renal cell carcinoma: a Surveillance, Epidemiology, and End Results [SEER]-Medicare-linked population study. Radiology.

[36] Cheung DC, Finelli A (2017). Active surveillance in small renal masses in the elderly: a literature review. Eur Urol Focus.

[37] Cella D, Grünwald V, Escudier B (2019). Patient-reported outcomes of patients with advanced renal cell carcinoma treated with nivolumab plus ipilimumab versus sunitinib (CheckMate 214): a randomised, phase 3 trial. Lancet Oncol.

[38] Sheng IY, Ornstein MC (2020). Ipilimumab and nivolumab as first-line treatment of patients with renal cell carcinoma: the evidence to date. Cancer Manag Res.

[39] Vitale MG, Scagliarini S, Galli L (2018). Efficacy and safety data in elderly patients with metastatic renal cell carcinoma included in the nivolumab Expanded Access Program (EAP) in Italy. PLoS One.

